# A Non-Cooperative Satellite Feature Point Selection Method for Vision-Based Navigation System

**DOI:** 10.3390/s18030854

**Published:** 2018-03-14

**Authors:** Mingfeng Ning, Shijie Zhang, Shiqiang Wang

**Affiliations:** Research Center of Satellite Technology, Harbin Institute of Technology, Harbin 150080, China; sjzhang@hit.edu.cn (S.Z.); wangshiqiang509@163.com (S.W.)

**Keywords:** non-cooperative satellite, feature point selection, vision-based navigation system, quasi-optimal method

## Abstract

The number of feature points on the surface of a non-cooperative target satellite used for monocular vision-based relative navigation affects the onboard computational load. A feature point selection method called the quasi-optimal method is proposed to select a subset of feature points with a good geometric distribution. This method, with the assumption that all of the feature points are in a plane and have the same variance, is based on the fact that the scattered feature points can provide higher accuracy than that of them grouped together. The cost is defined as a function of the angle between two unit vectors from the projection center to feature points. The redundancy of a feature point is calculated by summing all costs associated with it. Firstly, the feature point with the most redundant information is removed. Then, redundancies are calculated again with the second feature point removed. The procedures above are repeated until the desired number of feature points is reached. Dilution of precision (DOP) represents the mapping relation between the observation variance and the estimated variance. In this paper, the DOP concept is used in a vision-based navigation system to verify the performance of the quasi-optimal method. Simulation results demonstrate the feasibility of calculating the relative position and attitude by using a subset of feature points with a good geometric distribution. It also shows that the feature points selected by the quasi-optimal method can provide a high accuracy with low computation time.

## 1. Introduction

Relative navigation of a non-cooperative target satellite is an important part of space missions such as space offense and defense, on-orbit maintenance and orbital debris removal [1,2]. Different from the relative navigation of a cooperative target in which special optical markers are equipped on the target [3,4], the non-cooperative target has no preset special marker to be used. Since satellites are artificial objects, they usually have obvious feature points such as edges and corners on their surfaces, and these feature points can be used to obtain the relative navigation information [5]. If all of the feature points extracted from the surface of the target satellite are used for the relative navigation, the computational load will be very large. It is a great challenge for the chaser satellite with limited computing ability or a high real-time requirement. Therefore, a compromise between the computational load and the performance should be explored in practice. From this point of view, a subset of feature points can be selected for the relative navigation. The number of selected feature points should be far less than the number of the total feature points to reduce the computational load. Meanwhile, the accuracy with the selected feature points should meet the navigation requirement.

The estimated variance in a vision-based navigation system is closely related to the geometric distribution of feature points [6]. The accuracy can be described by the dilution of precision (DOP), which is widely used in the Global Navigation Satellite System (GNSS) [7,8,9]. A smaller DOP value indicates a higher navigation accuracy. For a vision-based navigation system that estimates position and attitude simultaneously, the geometric distribution of feature points affects the accuracies of position and attitude. Therefore, the accuracies of position and attitude can be described by position DOP (PDOP) and attitude DOP (ADOP), respectively.

The DOP of the vision-based system have been studied in recent years. Baine [10] studied the DOP of the vision-based system using the directional cosine matrix in the navigation frame and applied it to the consistency test. Park [11] studied the DOP of a combined vision and IMU system taking into account the alignment error and the mapping error, then analyzed the characteristics of the DOP in this system. Won [12] derived the weighted DOP using the unit vectors of feature points when the variances of feature points were different with respect to the geometrical distortion of the vision sensor. These research works were concerned with the DOP of absolute navigation and took the target body frame as the reference frame. To estimate the position and attitude of the non-cooperative target satellite, the DOP for the relative navigation in the sensor frame should be studied.

With the concept of DOP, the problem of the feature point selection for the non-cooperative target satellite can be described as selecting *m* from *n* feature points to make the DOP value as small as possible, where *m* represents the number of selected feature points and *n* represents the number of the total feature points. The most straightforward method called the optimal method [13] is to calculate the DOP values of all possible combinations with the number of *m*, then the subset with the minimum DOP value is selected. In this method, all possible combinations of *m* from *n* are tested, and each combination has matrix multiplication and inversion. The corresponding number of combinations is n!/[m!(n−m)!]. For example, if n=60 and m=15, then there will be almost $5.3\times 10^{13}$ combinations to be tested. The number of combinations rapidly increases with the increase of the total feature points, and it is unacceptable for the chaser satellite to complete the computation.

The feature point selection in a vision-based navigation system is similar to the satellite selection in GNSS. In GNSS, more and more satellites are in view because of the advent of Galileo, Compass and GPS. The increase of satellites will make use of the optimal method intractable. Many scholars have proposed different satellite selection methods in GNSS including the recursive method [14], maximum volume method [15,16], four-step method [17], neural network method [18,19] and other methods [20,21,22,23]. Since these methods utilize the characteristics of satellite orbit such as longitude and latitude or have a restriction on the selected number, they cannot be used to select feature points in a vision-based navigation system.

The image cannot be used to select feature points in different planes directly because of the lack of three-dimensional information. This paper assumes that feature points are in a plane. They can be easily obtained at close range navigation or based on the structure model of the target satellite, which is known in advance. Thus, the distribution of the feature points on the surface of the target satellite is similar to the distribution of the feature points on the image. The feature point selection is to select feature points on the image.

Inspired by Park [24], a quasi-optimal method is proposed for selecting feature points. In this method, the redundancy is determined by as a function of the angles between two unit vectors from the projection center to feature points, and the feature point with the largest redundancy is removed one by one. The quasi-optimal method yields a near-optimal geometric distribution without restrictions on the number of selected feature points and reduces the computational load significantly.

This paper is organized as follows. In Section 2, we review coordinate systems and the observation model. Section 3 gives the DOP of the vision-based navigation system in the sensor frame, and Section 4 gives the quasi-optimal feature point selection method. The performance of the quasi-optimal method is discussed in Section 5. Finally, Section 6 is the conclusion.

## 2. Vision Measurement Model

Relative position and attitude between two satellites are estimated with feature points on the surface of the target satellite. These feature points are projected on the image of the vision sensor equipped on the chaser satellite. Feature points are extracted from the image by extraction methods such as SIFT (scale-invariant feature transform) [25] or SURF (speeded up robust features) [26], and they are compared with feature points extracted from the next image to find the correspondences. A robust extraction and estimation process must be achieved in cases where the target satellite disappears from the vision sensor field of view [27]. Then, the relative position and attitude are estimated with the navigation algorithm based on these feature points.

### 2.1. Coordinate Systems

During the navigation, feature points are described in different coordinate systems. To obtain the observation model, the target body frame $O_{t}x_{t}y_{t}z_{t}$, the sensor frame $O_{s}x_{s}y_{s}z_{s}$ and the image frame $O_{im}xy$ are defined. Without loss of generality, we assume the sensor frame as the reference frame [28].

The origin of the image frame is the center of the image, and its *x* axis and *y* axis are parallel to the image’s row and column, respectively. The $z_{s}$ axis of the sensor frame, whose origin is the projection center of the vision sensor, is parallel to the projection axis and points to the target satellite. The $x_{s}$ axis and $y_{s}$ axis of the sensor frame are parallel to the *x* axis and *y* axis of the image frame, respectively. The distance between the image plane and the projection center is the focal length. The target body frame is fixed on the target satellite. Its origin and axes are defined based on the structure of the target satellite.

### 2.2. Observation Model

**Figure 1:**
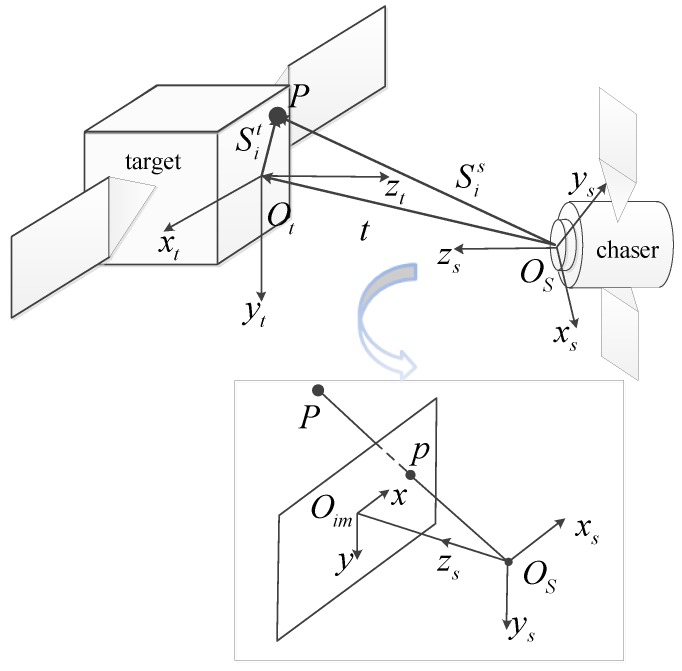
Coordinate systems.

As seen in Figure 1, the vector $S_{i}^{s}={\left[x_{i}^{s}\;\;y_{i}^{s}\;\;z_{i}^{s}\right]}^{T}$ from the projection center to the *i*-th feature point in the sensor frame can be expressed as:

(1) $$S_{i}^{s}=C_{t}^{s}S_{i}^{t}+t$$

where $S_{i}^{t}={\left[x_{i}^{t}\;\;y_{i}^{t}\;\;z_{i}^{t}\right]}^{T}$ is the position of the *i*-th feature point in the target body frame and $t=\;{\left[t_{x}\;\;t_{y}\;\;t_{z}\right]}^{T}$ is the position of the origin of the target body frame in the sensor frame. $C_{t}^{s}$ is the direction cosine matrix from the target body frame to the sensor frame, and it can be expressed as:

(2) $$C_{t}^{s}=\left[\begin{array}{c} \;\;\;\;\cos\left(\theta \right)\cos\left(\psi \right)\;\;\;\;\;\;\;\;\;\;\;\cos\left(\theta \right)\sin\left(\psi \right)\;\;\;\;\;\;\;\;\;\;-\sin\left(\theta \right)\;\\ -\cos\left(\varphi \right)\sin\left(\psi \right)+\sin\left(\varphi \right)\sin\left(\theta \right)\cos\left(\psi \right)\;\cos\left(\varphi \right)\cos\left(\psi \right)+\sin\left(\varphi \right)\sin\left(\theta \right)\sin\left(\psi \right)\;\sin\left(\varphi \right)\cos\left(\theta \right)\\ \sin\left(\varphi \right)\sin\left(\psi \right)+\cos\left(\varphi \right)\sin\left(\theta \right)\cos\left(\psi \right)\;-\sin\left(\varphi \right)\cos\left(\psi \right)+\cos\left(\varphi \right)\sin\left(\theta \right)\sin\left(\psi \right)\;\cos\left(\varphi \right)\cos\left(\theta \right) \end{array}\right]$$

where φ, θ and ψ represent roll angle, yaw angle and pitch angle, respectively. The vector $ȷ={\left[\varphi \;\;\theta \;\;\psi \right]}^{T}$ represents the rotating angle from the target body frame to the sensor frame.

According to the pinhole model, the measurement $z_{i}={\left[x_{i}\;\;y_{i}\right]}^{T}$ is the coordinate of the *i*-th feature point in the image frame. Thus, the relationship between $z_{i}$ and $S_{i}^{s}={\left[x_{i}^{s}\;\;y_{i}^{s}\;\;z_{i}^{s}\right]}^{T}$ can be obtained as:

(3) $$z_{i}=\left[\begin{array}{c} x_{i}\\ y_{i} \end{array}\right]=\frac{f}{z_{i}^{s}}\left[\begin{array}{c} x_{i}^{s}\\ y_{i}^{s} \end{array}\right]+v_{i}=\frac{f}{z_{i}^{s}}\left[\begin{array}{c} x_{i}^{s}\\ y_{i}^{s} \end{array}\right]+\left[\begin{array}{c} v_{xi}\\ v_{yi} \end{array}\right]$$

where *f* is the focal length of the vision sensor and $v_{i}={\left[v_{xi}\;v_{yi}\right]}^{T}$ is the measurement error of the *i*-th feature point.

Considering Equations (1) and (3) can be rewritten as:

(4) $$\left\{\begin{array}{c} x_{i}=f\frac{r_{11}x_{i}^{t}+r_{12}y_{i}^{t}+r_{13}z_{i}^{t}+t_{x}}{r_{31}x_{i}^{t}+r_{32}y_{i}^{t}+r_{33}z_{i}^{t}+t_{z}}\\ y_{i}=f\frac{r_{21}x{{}_{i}^{t}}_{t}+r_{22}y_{i}^{t}+r_{23}z_{i}^{t}+t_{y}}{r_{31}x_{i}^{t}+r_{32}y_{i}^{t}+r_{33}z_{i}^{t}+t_{z}} \end{array}\right.$$

where $r_{11}-r_{33}$ are the corresponding elements of $C_{t}^{s}$.

Equation (4) shows the relationship between the measurement of a feature point and the relative pose including the position t and the attitude ȷ. Let $h_{i}\left(t,ȷ\right)$ represent the nonlinear relationship, then $z_{i}$ can be rewritten as:

(5) $$z_{i}=\frac{f}{z_{i}^{s}}\left[\begin{array}{c} x_{i}^{s}\\ y_{i}^{s} \end{array}\right]+v_{i}=h_{i}\left(t,\;ȷ\right)+v_{i}$$

Defining $x={\left[t^{T}\;{ȷ}^{T}\right]}^{T}$, the first-order Taylor expansion of Equation (5) at $x_{0}={\left[t_{0}^{T}\;{ȷ}_{0}^{T}\right]}^{T}$ can be expressed as:

(6) $$z_{i}\approx h_{i}\left(t_{0}\;,{ȷ}_{0}\;\right)+\frac{\partial h_{i}}{\partial t^{T}}\left(t-t_{0}\right)+\frac{\partial h_{i}}{{\partial ȷ}^{T}}\left(ȷ-{ȷ}_{0}\right)+v_{i}$$

Considering Equation (6), the relation of the measurement residual $\delta z_{i}=z_{i}-h_{i}\left(t_{0}\;,{ȷ}_{0}\;\right)$, the relative position error $\delta t=t-t_{0}$ and the relative attitude error $\delta ȷ=ȷ-{ȷ}_{0}$ can be expressed as:

(7) $$\delta z_{i}=\left[\frac{\partial h_{i}}{\partial t^{T}}\;\;\frac{\partial h_{i}}{\partial {ȷ}^{T}}\right]\left[\begin{array}{c} \delta t\\ \delta ȷ \end{array}\right]+v_{i}=H_{i}\left[\begin{array}{c} \delta t\\ \delta ȷ \end{array}\right]+v_{i}$$

where:

$$\begin{array}{cc} \frac{\partial h_{i}}{\partial t^{T}} & =\frac{f}{z_{i}^{s}{\;}^{2}}\left[\begin{array}{c} z_{i}^{s}\;\;\;\;\;0\;\;-x_{i}^{s}\\ z_{i}^{s}\;\;\;-y_{i}^{s}\;\;\;0 \end{array}\right]\\ \frac{\partial h_{i}}{\partial {ȷ}^{T}} & =\frac{f}{z_{i}^{s}{\;}^{2}}\left[\begin{array}{c} \frac{\partial x_{i}^{s}}{\partial \phi }z_{i}^{s}-\;\frac{\partial z_{i}^{s}}{\partial \phi }x_{i}^{s}\;\;\frac{\partial x_{i}^{s}}{\partial \theta }z_{i}^{s}-\;\frac{\partial z_{i}^{s}}{\partial \theta }x_{i}^{s}\;\frac{\partial x_{i}^{s}}{\partial \psi }z_{i}^{s}-\;\frac{\partial z_{i}^{s}}{\partial \psi }x_{i}^{s}\\ \frac{\partial y_{i}^{s}}{\partial \phi }z_{i}^{s}-\;\frac{\partial z_{i}^{s}}{\partial \phi }y_{i}^{s}\;\;\frac{\partial y_{i}^{s}}{\partial \theta }z_{i}^{s}-\;\frac{\partial z_{i}^{s}}{\partial \theta }y_{i}^{s}\;\frac{\partial y_{i}^{s}}{\partial \psi }z_{i}^{s}-\;\frac{\partial z_{i}^{s}}{\partial \psi }y_{i}^{s} \end{array}\right]\\ \frac{\partial x_{i}^{s}}{\partial \varphi } & =0\\ \frac{\partial x_{i}^{s}}{\partial \theta } & =-\sin\left(\theta \right)\left(\cos\left(\psi \right)x_{i}^{t}+\sin\left(\psi \right)y_{i}^{t}\right)-\cos\left(\theta \right)z_{i}^{t}\\ \frac{\partial x_{i}^{s}}{\partial \psi } & =-\cos\left(\theta \right)\left(\sin\left(\psi \right)x_{i}^{t}-\cos\left(\psi \right)y_{i}^{t}\right)\\ \frac{\partial y_{i}^{s}}{\partial \varphi } & =\sin\left(\varphi \right)\left(\sin\left(\psi \right)x_{i}^{t}-\cos\left(\psi \right)y_{i}^{t}\right)+\cos\left(\varphi \right)\left(\sin\left(\theta \right)\left(\cos\left(\psi \right)x_{i}^{t}+\sin\left(\psi \right)y_{i}^{t}\right)+\cos\left(\theta \right)z_{i}^{t}\right)\\ \frac{\partial y_{i}^{s}}{\partial \theta } & =\sin\left(\varphi \right)\left(\cos\left(\theta \right)\left(\cos\left(\psi \right)x_{i}^{t}+\sin\left(\psi \right)y_{i}^{t}\right)-\sin\left(\theta \right)z_{i}^{t}\right)\\ \frac{\partial y_{i}^{s}}{\partial \psi } & =-\cos\left(\varphi \right)\left(\cos\left(\psi \right)x_{i}^{t}+\sin\left(\psi \right)y_{i}^{t}\right)+\sin\left(\varphi \right)\sin\left(\theta \right)\left(-\sin\left(\psi \right)x_{i}^{t}+\cos\left(\psi \right)y_{i}^{t}\right)\\ \frac{\partial z_{i}^{s}}{\partial \varphi } & =\cos\left(\varphi \right)\left(\sin\left(\psi \right)x_{i}^{t}-\cos\left(\psi \right)y_{i}^{t}\right)-\sin\left(\varphi \right)\left(\sin\left(\theta \right)\left(\cos\left(\psi \right)x_{i}^{t}+\sin\left(\psi \right)y_{i}^{t}\right)+\cos\left(\theta \right)z_{i}^{t}\right)\\ \frac{\partial z_{i}^{s}}{\partial \theta } & =\cos\left(\varphi \right)\left(\cos\left(\theta \right)\left(\cos\left(\psi \right)x_{i}^{t}+\sin\left(\psi \right)y_{i}^{t}\right)-\sin\left(\theta \right)z_{i}^{t}\right)\\ \frac{\partial z_{i}^{s}}{\partial \psi } & =\sin\left(\varphi \right)\left(\cos\left(\psi \right)x_{i}^{t}+\sin\left(\psi \right)y_{i}^{t}\right)+\cos\left(\varphi \right)\sin\left(\theta \right)\left(-\sin\left(\psi \right)x_{i}^{t}+\cos\left(\psi \right)y_{i}^{t}\right) \end{array}$$

## 3. DOP in a Vision-Based Navigation System

In a vision-based navigation system, the geometric distribution of feature points affects the navigation accuracy. The DOP is determined and utilized to estimate the accuracy of the vision-based navigation system.

For n feature points, δz is the measurement residual vector with 2n×1 dimensions and H is the mapping matrix after being linearized with 2n×6 dimensions. To determine the DOP in a vision-based navigation system, the error of the state vector $\delta x={\left[\delta t^{T}\;\delta {ȷ}^{T}\right]}^{T}$ should be estimated assuming that δz and H are known. The least square method is used to solve Equation (7), and the error function $J\left(\delta x\right)$ is defined as:

(8) $$J\left(\delta x\right)={\left(\delta z-H\delta x\right)}^{T}\left(\delta z-H\delta x\right)$$

where $J\left(\delta x\right)$ is a quadratic form and can be expanded as:

(9) $$\begin{array}{cc} J\left(\delta x\right) & =\delta z^{T}\delta z-\delta z^{T}H\delta x-\delta x^{T}H^{T}\delta z+\delta x^{T}H^{T}H\delta x\\ & =\delta z^{T}\delta z-2\delta z^{T}H\delta x+\delta x^{T}H^{T}H\delta x \end{array}$$

Taking the derivative of $J\left(\delta x\right)$ with respect to δx, we can get:

(10) $$\frac{\partial J\left(\delta x\right)}{\partial \delta x}=-2H^{T}\delta z+2H^{T}H\delta x$$

To get the optimal δx, Equation (10) should be equal to 0. Thus, it can be expressed as:

(11) $$-2H^{T}\delta z+2H^{T}H\delta x=0$$

By solving Equation (11), δx is estimated as:

(12) $$\delta x={\left(H^{T}H\right)}^{-1}H^{T}\delta z$$

Using Equation (12), the covariance of the state vector δx can be expressed as:

(13) $$\begin{array}{cc} E\left[\delta x\delta x^{T}\right] & =E\left[{\left(H^{T}H\right)}^{-1}H^{T}\delta z\delta z^{T}H{\left(H^{T}H\right)}^{-1}\right]\;\\ & ={\left(H^{T}H\right)}^{-1}H^{T}E\left[\delta z\delta z^{T}\right]H{\left(H^{T}H\right)}^{-1} \end{array}$$

It is assumed that all measurements are independent with the same variance $\sigma_{v}^{2}$. The horizontal and vertical variances of a feature point are also assumed to be independent, although they are measured simultaneously. Thus, the variance δz can be described as $E\left[\delta z\delta z^{T}\right]=\sigma_{v}^{2}$, and Equation (13) can be simplified as:

(14) $$E\left[\delta x\delta x^{T}\right]={\left(H^{T}H\right)}^{-1}\sigma_{v}^{2}$$

In Equation (14), the matrix ${\left(H^{T}H\right)}^{-1}$ represents the mapping relationship between the measurement variance and the state error variance. $E\left[\delta x\delta x^{T}\right]$ is a diagonal matrix because of the independence of measurements. Thus, DOP is defined as the sum of the elements along the main diagonal of A with $A={\left(H^{T}H\right)}^{-1}$.

Different from the state vector in the GNSS, which has only the term of position, the state vector of the vision-based navigation system takes position and attitude into consideration. Thus, the DOP of the vision-based navigation system can be divided into PDOP and ADOP, and they are defined as:

(15) $$\begin{array}{c} PDOP=\sqrt{A_{11}+A_{22}+A_{33}}\\ ADOP=\sqrt{A_{44}+A_{55}+A_{66}} \end{array}$$

Since the DOP of the vision-based navigation system includes PDOP and ADOP, the optimal method for the system searches the subset with the minimum PDOP value or the minimum ADOP value. Subsets with small PDOP values tend to have good geometric distributions. Therefore, they usually have small ADOP values and vice versa.

Equations (14) and (15) show that a smaller PDOP (or ADOP) means a higher navigation accuracy of position (or attitude). In GNSS, DOP becomes smaller as the number of satellites increases. It is always true that the PDOP (or ADOP) of the vision-based navigation system will become smaller when more feature points are selected.

## 4. Quasi-Optimal Method for Selecting Feature Points

A large number of feature points can be extracted from the image. This will result in a large computational load to finish the calculation if all feature points are used. Therefore, it is necessary to select a subset of feature points. The geometric distribution of the feature points on the surface of the target satellite plays a key role in the navigation accuracy. Assuming that feature points are in a plane, this section presents the quasi-optimal method for selecting a subset of feature points.

### 4.1. The Quasi-Optimal Method

The quasi-optimal method [24] is initially used to select satellites from different constellations in GNSS. The elements of the matrix **H** are the direction cosines of angles between the coordinate axis and vectors from the receiver to satellites. Therefore, the matrix **H** is used to select satellites directly. In a vision-based navigation system, the matrix **H** has no physical meaning. Thus, other measurements are needed to select feature points.

**Figure 2:**
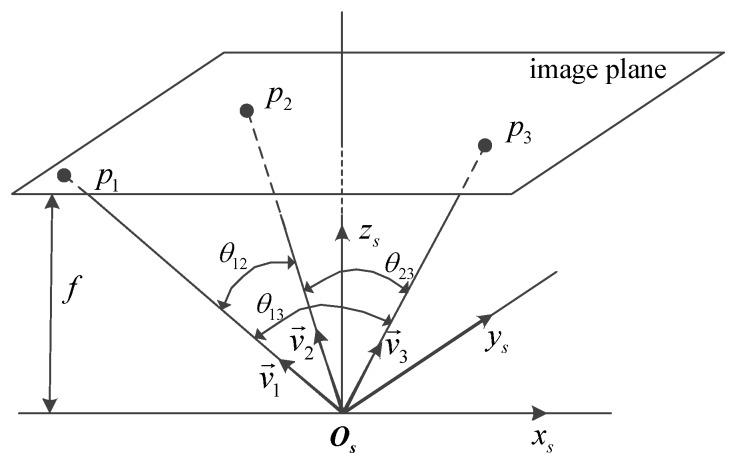
The relationship between feature points, unit vectors and angles

The distribution of the feature points on the image can be described by the angles between the unit vectors from the projection center to feature points as shown in Figure 2. A small angle between two unit vectors indicates the closeness of the two corresponding feature points on the image.

The cost used in this method is inspired by the intuitive concept that two unit vectors close to each other provide more redundant information. The cost $J_{ij}$ for the *i*-th unit vector and the *j*-th unit vector is defined as:

(16) $$J_{ij}=\cos2\theta_{ij}$$

where $\theta_{ij}$ is the angle between the *i*-th unit vector and the *j*-th unit vector.

The cost is a function of the angle between two unit vectors. If the angle between two unit vectors is small or the corresponding two feature points on the image are close to each other, the cost will be large. Otherwise, the cost will be small. The redundancy of the *i*-th unit vector can be defined as the sum of costs between the *i*-th unit vector and the other unit vectors. It can be described as:

(17) $$J_{i}=\sum_{j=1}^{n}\left(\cos2\theta_{ij}\right)$$

where *n* is the number of the total feature points. Equation (17) represents the redundancy degree of the *i*-th unit vector. Redundancies of other unit vectors can be determined in the same way.

For the *i*-th feature point, its unit vector is represented as $v_{i}$. Thus, $K={\left[v_{1}\;v_{2}\;\cdots \;v_{n}\right]}^{T}$ represents the unit vectors of *n* feature points. It is an n×3 matrix, and the matrix D can be calculated as:

(18) $$D=K^{T}K=\left[\begin{array}{c} \cos\theta_{11}\;\;\cos\theta_{12}\;\;\ldots \;\;\cos\theta_{1n}\\ \cos\theta_{21}\;\;\cos\theta_{22}\;\;\ldots \;\;\cos\theta_{2n}\\ \;\;\;\;\vdots \;\;\;\vdots \;\;\;\;\;\vdots \\ \cos\theta_{n1}\;\;\cos\theta_{n2}\;\;\ldots \;\;\cos\theta_{nn} \end{array}\right]$$

where the element of D, $d_{ij}$, is the cosine of the angle between the *i*-th unit vector and the *j*-th unit vector. With Equation (18), the redundancy of the *i*-th unit vector can be rewritten as:

(19) $$\begin{array}{cc} J_{i}^{1}=\sum_{j=1}^{n}\left(\cos2\theta_{ij}\right) & =\sum_{j=1}^{n}\left(2\cos^{2}\left(\theta_{ij}\right)-1\right)\\ & =\sum_{j=1}^{n}\left(2d_{ij}^{2}-1\right) \end{array}$$

where the superscript 1 indicates that the redundancy lies in initial redundancies.

The redundancy of the *i*-th unit vector can be expressed in terms of the sum of the squares of all elements in the *i*-th row of the matrix D. All redundancies can be calculated, and the unit vector with largest redundancy can be determined as:

(20) $$J_{k_{1}}^{1}=\max\left\{J_{1}^{1},J_{2}^{1},\cdots ,J_{n}^{1}\right\}$$

where the subscript $k_{1}$ represents the index of the feature point with the largest redundancy.

The $k_{1}^{th}$ feature point should be removed because it provides the least additional information. The $k_{1}$-th row and the $k_{1}$-th column are deleted from the matrix D, and a new matrix D with $\left(n-1\right)\times \left(n-1\right)$ dimensions is generated.

Then, new redundancies and the largest redundancy $J_{k_{2}}^{2}$ are recalculated for the new matrix D using Equation (21). Remove the $k_{2}$-th feature point, and delete the $k_{2}$-th row and column from the new D. Another new matrix D with $\left(n-2\right)\times \left(n-2\right)$ dimensions is generated.

(21) $$\begin{array}{cc} J_{i}^{2} & =\sum_{j=1}^{n-1}\left(2d_{ij}^{2}-1\right)\\ J_{k_{2}}^{2} & =\max\left\{J_{1}^{2},J_{2}^{2},\cdots ,J_{n}^{2}\right\} \end{array}$$

In order to reduce the computational load, Equation (22) is used to compute new redundancies by subtracting the costs of the previous redundancies associated with the removed feature point.

(22) $$J_{i}^{2}=J_{i}^{1}-\left(2d_{ik_{1}}^{2}-1\right)$$

**Figure 3:**
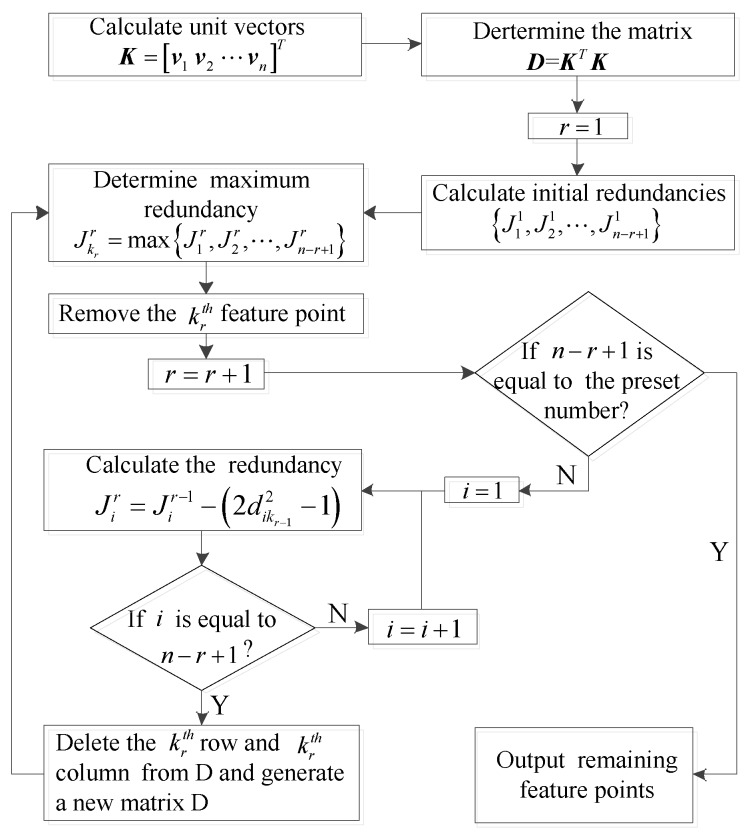
The flowchart of the quasi-optimal method

Repeat the procedures above until the number of remaining feature points is the same as the preset number. The flowchart of the method is shown in Figure 3.

### 4.2. Analysis of the Method

The quasi-optimal method proposed in this paper is an iterative method that removes the most redundant feature points one by one. An alternative to the quasi-optimal method is the one-step method. This method calculates the initial redundancies $\left\{J_{1}^{1},J_{2}^{1},\cdots ,J_{n}^{1}\right\}$ firstly. Then, it removes multiple feature points with larger redundancies directly, and the remaining feature points are the selection result.

The feature point with the largest redundancy has different effects on the redundancies of other feature points. Therefore, the feature point with the second largest redundancy in the initial redundancies is not usually the most redundant after the feature point with the largest redundancy is removed. The one-step method does not consider this effect, but the iterative method does. This is the reason why the iterative method is chosen in this paper to filter out feature points. To demonstrate the advantage of the iterative method, an example for selecting two from four feature points is given.

**Figure 4:**
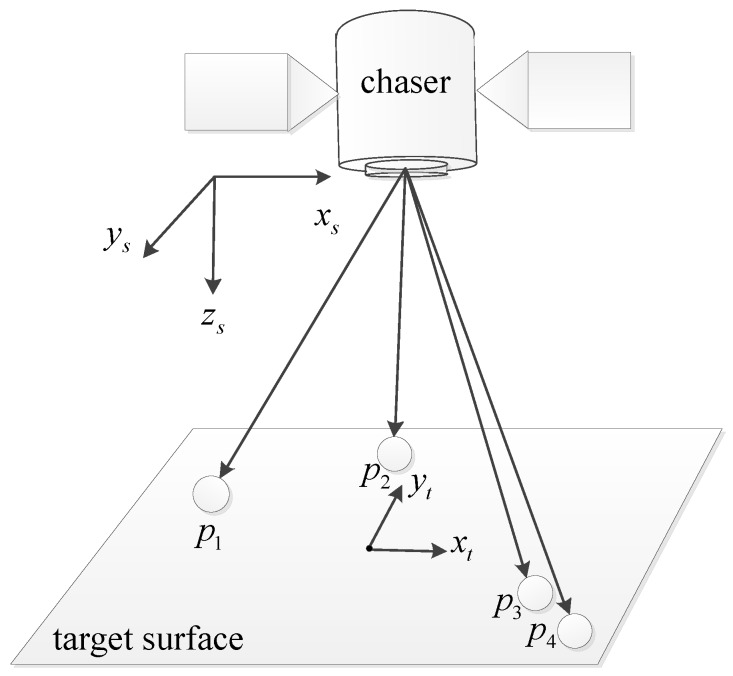
The distribution of the four feature points

In this example, it is assumed that only translation motion between the target satellite and the chaser satellite exists. There are four feature points on the surface of the target satellite, as shown in Figure 4, and two of them with a low PDOP value will be selected. The initial condition for this example is shown in Table 1. Coordinates of the four feature points are in the target body frame.

**Figure 5:**
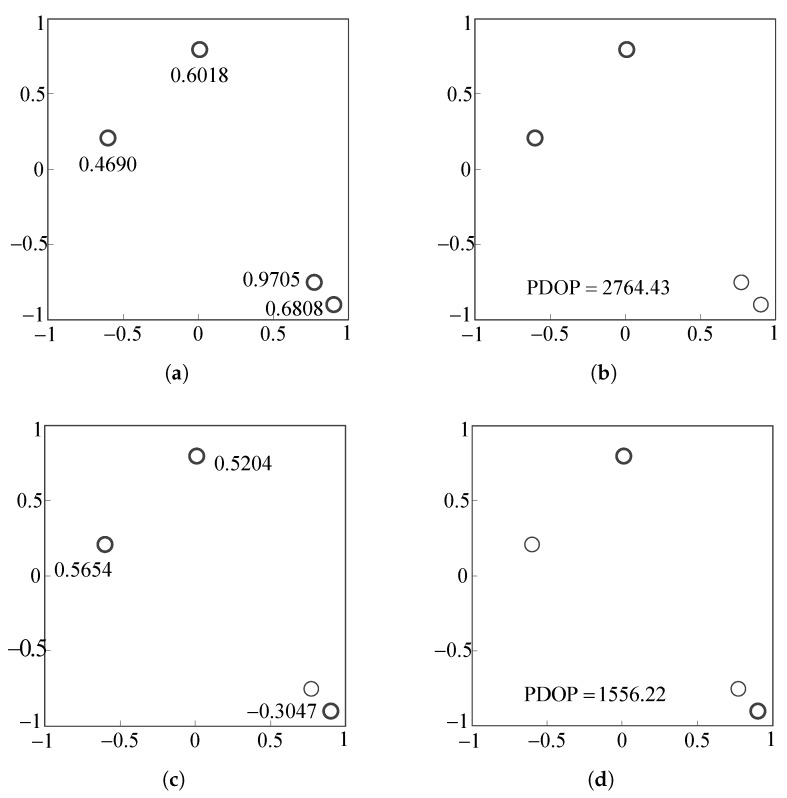
Processes of the iterative method and one-step method for selecting two out of four feature points. (**a**) Redundancies of the four feature points; (**b**) removing two feature points with larger redundancies by the one-step method; (**c**) redundancies after removing the feature point with the largest redundancy; (**d**) removing the second feature point based on new redundancies. PDOP, position dilution of precision.

Before removing feature points, the redundancies of the four feature points should be calculated first. The redundancies of $p_{3}$ and $p_{4}$ are larger than those of $p_{1}$ and $p_{2}$, as shown in Figure 5a. The one-step method is shown in Figure 5b. It removes the two feature points $p_{3}$ and $p_{4}$ directly because they have larger redundancies. The PDOP value of the remaining feature points, $p_{1}$ and $p_{2}$, is 2764.43, which is approximately 1.8-times greater than the optimal PDOP 1525.84, as shown in Table 2.

Figure 5c is the first step of the iterative method. It removes the feature point $p_{3}$ and recalculates the new redundancies of the remaining three feature points. Then, the feature point $p_{1}$ is removed because it has the largest redundancy in new redundancies as shown Figure 5d. The two feature points $p_{2}$ and $p_{4}$ are left. Their PDOP is 1556.22, which is only 2.0% higher than the optimal PDOP value.

In this example, the iterative method shows a better performance than the one-step method. As shown in Table 2, the angle between the unit vectors of $p_{3}$ and $p_{4}$ is only 4.874 degrees. Its cost is 0.9856, which is much larger than others. Thus, the redundancies of $p_{3}$ and $p_{4}$ may be larger than those of the others because of the existence of the cost $J_{34}$. When $p_{4}$ is removed, the cost $J_{34}$ does not exist, and the redundancy of $p_{4}$ is lower than the redundancy of others.

## 5. Simulation and Result

In order to verify the performance of the feature point selection method in the vision-based navigation system, the quasi-optimal method is compared with the optimal method in terms of accuracy and computation time in this section. Due to the significantly increase of the possible combinations, the optimal method could not be completed if the number of the total feature points is large. Therefore, the number of feature points in this section is small.

Simulations in this section are conducted with MATLAB running on a computer with 2.67 GHZ Intel(R) Core(TM)2 CPU and 4 GB RAM. Feature points used in simulations are generated by MATLAB randomly in a 1 m × 1 m square plane. Then, coordinates of these feature points in the sensor frame are determined utilizing the coordinate transformation. Using the pinhole model, feature points are projected on the image. The simulation condition is shown in Table 3.

### 5.1. Accuracy Evaluation

DOP values are used to evaluate the accuracy of the relative position and attitude with the selected feature points. For better analysis, DOP ratios are used to evaluate the performance. The DOP ratios, $\xi_{P}$ and $\xi_{A}$, are defined as:

(23) $$\begin{array}{c} \xi_{P}=\mathrm{PDO}P_{quasi}/\mathrm{PDO}P_{optimal}\\ \xi_{A}=\mathrm{ADO}P_{quasi}/\mathrm{ADO}P_{optimal} \end{array}$$

where subscripts *quasi* and *optimal* represent the DOP values of the quasi-optimal method and the optimal method.

Since the optimal method provides the smallest PDOP and ADOP values in all possible combinations, $\xi_{P}$ and $\xi_{A}$ are always greater than or equal to one. The closer $\xi_{P}$ and $\xi_{A}$ to one, the better the geometric distribution of feature points is.

#### 5.1.1. Simulations for Different Total Numbers

The simulations assume that there are four groups of feature points with different numbers (12, 14, 16 and 18), but only eight feature points are selected. In each group, feature points for 1000 cases are randomly generated. The two selection methods are implemented and tested with these feature points. For each case, the PDOP and ADOP values of the feature points selected by the quasi-optimal method are calculated. The smallest PDOP and ADOP values are selected from all possible PDOP and ADOP values. Then, the DOP ratios, $\xi_{P}$ and $\xi_{A}$, are determined and evaluated.

**Figure 6:**
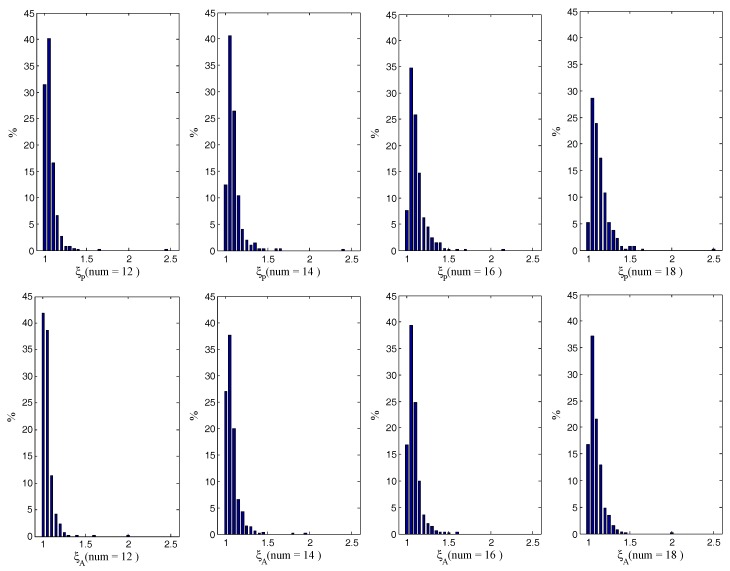
Distributions of DOP ratios for different numbers of total feature points.

The distributions of $\xi_{P}$ and $\xi_{A}$ for 1000 cases are evaluated in Figure 6 when the numbers of feature points are 12, 14, 16 and 18, respectively. The bin size is 0.05. It can been seen that the DOP ratios are near one, and most of them are less than 1.2. There are cases with PDOP ratios between 2 and 2.5 and ADOP ratios near two in the four groups. Although, the DOP ratios in these cases are a little large for navigation, this is acceptable because large DOP ratios account for an extremely low percentage.

The average and maximum DOP ratios for different numbers are shown in Table 4. In the group of 18 feature points, the average $\xi_{P}$ and $\xi_{A}$ are 1.1324 and 1.0897, respectively. Thus, the average PDOP value of the quasi-optimal method is 13.24% greater than the optimal PDOP value, and the average ADOP value of the quasi-optimal method is only 8.97% greater than the optimal ADOP value on average in this group. This indicates that the average PDOP and ADOP values of the selected feature points are close to the average optimal values. Moreover, the other groups perform better than this group on average values.

**Figure 7:**
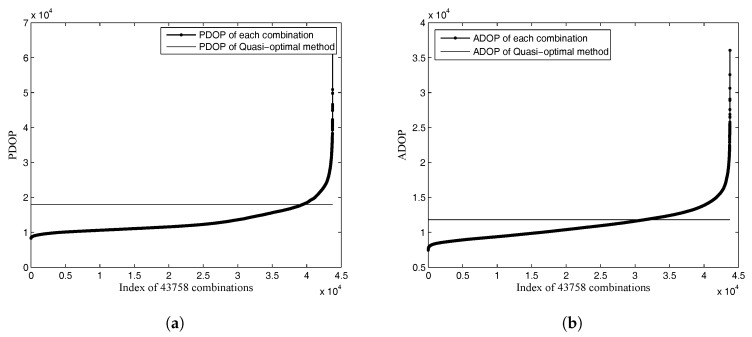
All possible DOP values and DOP values of the quasi-optimal method with $\xi_{P}=2.5185$ and $\xi_{A}=2.0216$. (**a**) PDOP values; (**b**) attitude DOP (ADOP) values.

Two cases in the group of 18 feature points are used to analyze the DOP values in worst cases. The $\xi_{P}$ and $\xi_{A}$ of the two cases are 2.5185 and 2.0216, respectively. They are maximum ratios in 1000 cases, as shown in Table 4. The DOP values of the quasi-optimal method and all possible DOP values are shown in Figure 7. There are 43,758 combinations, and all possible DOP values are sorted in ascending order. This shows that the PDOP and ADOP values of the quasi-optimal method in the two worst cases are not significantly large compared with those of all possible combinations.

#### 5.1.2. Simulations for Different Selected Numbers

To further evaluate the accuracy of the quasi-optimal method, the two methods are implemented in 1000 cases with 18 feature points. In this simulation, the number of selected feature points is assigned from 4–17 because it takes at least four feature points in a plane to make $H^{T}H$ a nonsingular matrix.

**Figure 8:**
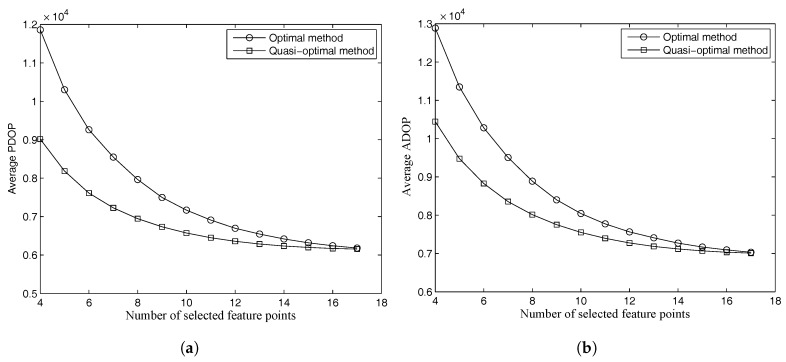
Average PDOP and ADOP values for different selected numbers of the two methods. (**a**) PDOP values; (**b**) ADOP values.

As shown in Figure 8, the DOP values of the two methods have a similar trend. The figure shows that the mathematical proof derived by Yarlagadda [29] for DOP in GNSS also works in a vision-based navigation system. The proof indicates that the DOP value decreases as the number of the selected measurements increases. The DOP values of the selected feature points approach those of all feature points when the number of selected feature points increases.

The average PDOP values decrease, and the ratios of the PDOP values given by the two methods to those of all feature points on average are shown in Table 5. The average PDOP value is flat if the decrease is less than 5%. As shown in Table 5, the PDOP value of the optimal method is flat when the number of selected feature points is eight. However, the quasi-optimal method requires more than 10 feature points to make the average PDOP value flat. The PDOP value of the optimal method is 9.55% bigger than that of all feature points when the selected number is nine. The quasi-optimal method can reach goodaccuracy only when the number is more than 11. This indicates that the position accuracy of the selected feature points is a little lower than that of all feature points with a reasonable selected number. The optimal method performs better than the quasi-optimal method. However, the quasi-optimal method can also perform well by adding the selected feature points. The same result can be obtained for ADOP or the attitude accuracy according to Table 6.

### 5.2. Time Performance

**Figure 9:**
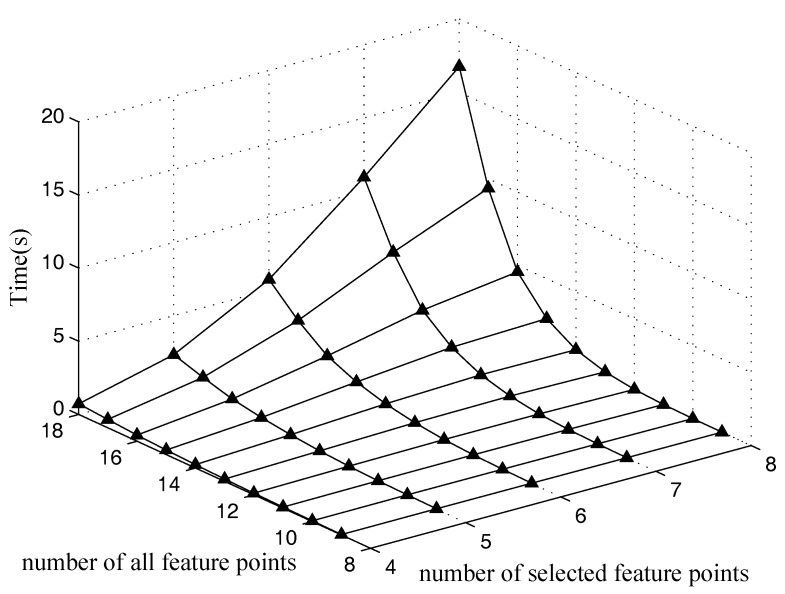
Time performance for the optimal method.

**Figure 10:**
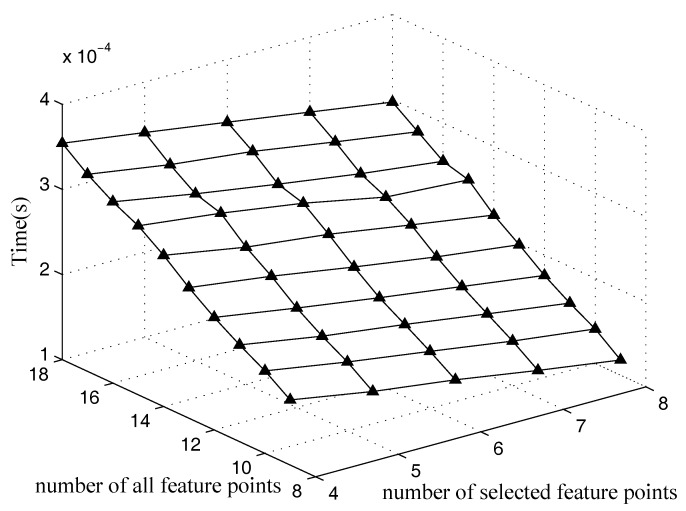
Time performance for the quasi-optimal method.

The average computation time of the optimal method and that of the quasi-optimal method are estimated in this section. The two methods are used to select 4–8 feature points out of 9–18 feature points for 1000 cases. Figure 9 shows the average time of the optimal method. The average time of the optimal method increases along with the increasing of the number of the total feature points. The reason is that more feature points provide more possible combinations. Figure 10 is the average time of the quasi-optimal method proposed in this paper. It shows that the time of the quasi-optimal method increases as the number of feature points increases. Notice that the time of the quasi-optimal is four orders of magnitude lower than that of the optimal method. This means that the quasi-optimal method outperforms the optimal method greatly in terms of computation time.

## 6. Conclusions

Relative navigation based on vision sensors increasingly becomes significant because of the advantage of accuracy. However, too many feature points on the surface of a target satellite will result in a large computational load and burden the chaser satellite. The main purpose of this paper is to provide an effective method to select feature points.

This paper proposes a quasi-optimal method for selecting a subset of feature points with a good geometric distribution. The method is an iterative method. Firstly, the initial redundancies are calculated, and the feature point with the largest redundancy is removed. Then, the redundancies of the remaining feature points are recalculated, and the second feature point is removed. Feature points are removed one by one through the procedures above until the desired number is reached. Moreover, the dilution of precision is used to verify the performance of the quasi-optimal method. Simulations show that the quasi-optimal method can select feature points with good geometric distribution. Although cases with large PDOP or ADOP values exist, they only account for an extremely low percentage. The results also show that the quasi-optimal method can perform well by adding the selected feature points and requires only a limited time for computation. 

## Figures and Tables

**Table 1:** Coordinates of feature points, focal length and relative position.

Parameter	Parameter Value
$p_{1}$ coordinate (m)	${\left[-0.6\;0.2\;0\right]}^{T}$
$p_{2}$ coordinate (m)	${\left[0\;\;0.8\;0\right]}^{T}$
$p_{3}$ coordinate (m)	${\left[0.8\;-0.8\;0\right]}^{T}$
$p_{4}$ coordinate (m)	${\left[0.9\;-0.9\;0\right]}^{T}$
sensor focal length (mm)	4.0
relative position (m)	${\left[0\;0\;2\right]}^{T}$

**Table 2:** Angles, costs and PDOP values for all possible combinations.

Combination	Angle	Cost	PDOP
$p_{1}p_{2}$	22.975	0.6953	2764.43
$p_{1}p_{3}$	43.958	0.0364	1667.95
$p_{1}p_{4}$	48.731	−0.1299	1525.84
$p_{2}p_{3}$	46.474	−0.0514	1716.95
$p_{2}p_{4}$	50.036	−0.1749	1556.22
$p_{3}p_{4}$	4.874	0.9856	9209.04

**Table 3:** Simulation parameters.

Parameter	Parameter Value
focal length (mm)	4.0
pixel size (μm)	8.9×8.9
image size	1280×768
relative position (m)	${\left[0.5\;1\;10\right]}^{T}$
relative attitude (${}^{\circ }$)	${\left[30\;10\;25\right]}^{T}$

**Table 4:** The statistics of DOP ratios.

Total Number	$\xi_{P}$		$\xi_{A}$	
	avg	max	avg	max
12	1.0642	2.4478	1.0502	1.9767
14	1.0964	2.3875	1.0748	1.9610
16	1.1156	2.1719	1.0862	1.5859
18	1.1324	2.5185	1.0897	2.0216

**Table 5:** The statistics of average PDOP values, decreases and ratios to all feature points’ PDOP values for the two methods.

Selected Number	Optimal Method			Quasi-Optimal Method		
	Avg PDOP	% Decrease	Ratio to All	Avg PDOP	% Decrease	Ratio to All
4	9015.95	−	1.4666	11857.30	−	1.9288
5	8183.07	9.2378	1.3311	10299.56	13.1374	1.6754
6	7612.24	6.9758	1.2383	9259.25	10.1005	1.5062
7	7227.73	5.0512	1.1757	8548.65	7.6744	1.3906
8	6945.25	3.9083	1.1298	7963.22	6.8482	1.2953
9	6734.66	3.0322	1.0955	7497.85	5.8441	1.2196
10	6573.73	2.3896	1.0693	7169.66	4.3771	1.1663
11	6451.26	1.8630	1.0494	6908.89	3.6371	1.1238
12	6357.54	1.4527	1.0342	6696.81	3.0697	1.0893
13	6286.47	1.1178	1.0226	6545.02	2.2667	1.0647
14	6235.02	0.8185	1.0142	6419.64	1.9157	1.0443
15	6197.87	0.5958	1.0082	6319.62	1.5580	1.0280
16	6172.17	0.4147	1.0040	6240.86	1.2462	1.0152
17	6155.72	0.2665	1.0013	6182.30	0.9383	1.0057
All	6147.58	−	−	6147.58	−	−

**Table 6:** The statistics of average ADOP values, decreases and ratios to all feature points’ ADOP for the two methods.

Selected Number	Optimal Method			Quasi-Optimal Method		
	Avg. ADOP	% Decrease	Ratio to All	Avg. ADOP	% Decrease	Ratio to All
4	10441.90	−	1.4921	12885.03	−	1.8412
5	9474.39	9.2656	1.3539	11347.85	11.9300	1.6216
6	8826.84	6.8348	1.2613	10282.25	9.3903	1.4693
7	8354.98	5.3457	1.1939	9507.36	7.5362	1.3586
8	8013.48	4.0874	1.1451	8888.22	6.5122	1.2701
9	7752.69	3.2544	1.1078	8399.48	5.4987	1.2003
10	7550.07	2.6134	1.0789	8043.02	4.2439	1.1493
11	7395.22	2.0511	1.0568	7772.89	3.3586	1.1107
12	7275.54	1.6183	1.0397	7565.00	2.6745	1.0810
13	7185.91	1.2319	1.0268	7409.61	2.0541	1.0588
14	7118.81	0.9338	1.0173	7271.70	1.8612	1.0391
15	7069.25	0.6961	1.0102	7169.49	1.4055	1.0245
16	7033.49	0.5059	1.0051	7093.89	1.0545	1.0137
17	7009.92	0.3351	1.0017	7032.07	0.8715	1.0049
All	6998.05	−	−	6998.05	−	−
